# The Neural Basis of Skull Vibration Induced Nystagmus (SVIN)

**DOI:** 10.3390/audiolres11040050

**Published:** 2021-10-14

**Authors:** Ian S. Curthoys

**Affiliations:** Vestibular Research Laboratory, School of Psychology, The University of Sydney, Sydney, NSW 2006, Australia; ian.curthoys@sydney.edu.au

**Keywords:** vestibular, otolith, utricular, saccular, labyrinth, VEMP, semicircular canal

## Abstract

I list a summary of the major clinical observations of SVIN in patients with total unilateral vestibular loss (TUVL) and show how basic results from neurophysiology can explain these clinical observations. The account integrates results from single neuron recordings of identified semicircular canal and otolith afferent neurons in guinea pigs in response to low frequency skull vibration with evidence of the eye movement response in cats to selective semicircular canal stimulation (both individual and combined) and a simple model of nystagmus generation to show how these results explain most of the major characteristics of SVIN.

## 1. Introduction

Skull vibration induced nystagmus (SVIN) is a dramatic clinical observation—if a vibrator is applied to either mastoid of a patient with a total unilateral vestibular loss (TUVL) and switched on at a frequency of 100 Hz and at moderate intensity (about the strength a body massager) the patient immediately has a nystagmus, mainly horizontal, with the clinically obvious quick phases (QPs) beating away from the lesioned ear [1]. The nystagmus ceases at the offset of the vibration with no afternystagmus. Video recordings show the nystagmus is comprised of slow phase eye velocity (SPV) deviations away from the healthy ear, interspersed with oppositely directed quick return phases (QPs) away from the lesioned ear. The SPV and QP are driven by different neural mechanisms [2]. The quick phases are easily detectable by the clinician at the bedside (with Frenzel glasses) but quantifying the slow phase eye velocity requires three dimensional recordings (horizontal, vertical, and torsional) of the eye movement by scleral search coils or video as shown in Figure 1 from [1].

With SVIN, the startling result is that the direction of the nystagmus is the same for stimulation of either mastoid! As a control: the same procedure in healthy subjects does not elicit such a consistent nystagmus with slow phase eye velocity above 2.5 deg/s [1]. The first question is what vestibular receptors are being activated by mastoid vibration, and the physiological evidence for that is summarized below [3,4]. The second question is why such activation results in nystagmus and a simple, evidence-based, “imbalance model” of nystagmus generation is presented. The third question is to explain the exact direction of the slow phase eye velocity component of the nystagmus and to do this I refer to the results of selective stimulation of nerves from single canals (or combination of canal nerves [5]). These data are used to explain the results from 3-D eye movement measurement of SVIN in different categories of human testing. Many characteristics of SVIN have been documented by Dumas and his colleagues [1,6,7,8] and the main features are summarized in Table 1.

## 2. Vibration—Physiological Evidence

**Figure 2:**
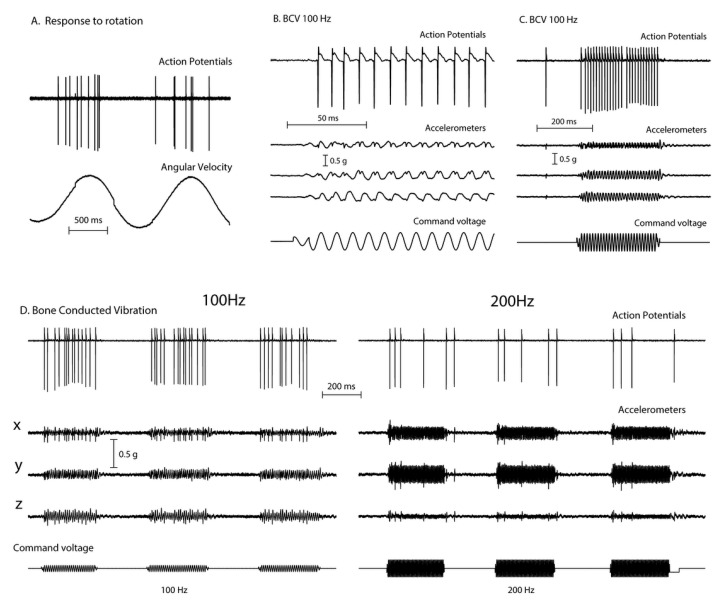
The responses (action potentials) of a primary semicircular canal neuron to low frequency skull vibration. (**A**) Responses to angular acceleration, demonstrating that this is a semicircular canal neuron. (**B**) In response to 100Hz vibration, the neuron fires one action potential per cycle and it is phase locked to the stimulus, as shown by the small spikes in the accelerometer records and the very even spacing of action potentials in (**C**). However, if frequency is increased the neural response decreases (**D**) even when the vibration intensity is increased (as shown by the smaller response at 200Hz compared to 100 Hz). Reproduced from [2] with permission of Wolters Kluwer Health.

Recordings from single vestibular afferents in guinea pigs have shown that low frequency skull vibration (100 Hz) is an effective stimulus activating those semicircular canal and otolith neurons with irregular resting discharge (Figure 2). These afferents are called “irregular” because in these neurons the interval between action potentials in the absence of stimulation—the resting discharge—is variable and thus termed “irregular”. These irregular afferents innervate the amphora shaped type I receptors at the crest of the crista or at the striola of the otolithic maculae [9,10] and these afferents are activated with high sensitivity by 100 Hz vibration. In contrast other afferent neurons, synapsing predominantly on type II receptors on the slopes of the crista or in the extrastriolar area of the otolithic maculae, show a regular resting discharge but have a very poor or absent response to vibration at clinically safe intensities [4].

These activated irregular neurons typically have low resting rates and some even have zero resting rate [11,12,13]. When activated by 100Hz vibration, the irregular canal afferents are activated and at 100Hz, fire one action potential per cycle (Figure 2B,C), synchronized with the stimulating frequency. This synchronization shows that each cycle of the vibration is the effective stimulus for the receptor and afferent, and it explains why the SPV of SVIN depends on vibration frequency (at least up to 100 Hz). A low frequency stimulus (e.g., 30 Hz) only elicits a modest phase-locked neural firing rate (30 spikes/s) whereas a high frequency stimulus (100 Hz) elicits a much higher firing rate (100 spikes/s). Increasing stimulus frequency above about 100 Hz is progressively less effective (Figure 2D), probably because the stimulus intensity at clinically acceptable stimulus levels is not sufficient to activate the receptors.

Phase-locking of canal neurons to the vibration stimulus explains the abrupt stimulus-locked onset and offset of SVIN without any afternystagmus. In TUVL patients the unilateral 100 Hz mastoid vibration stimulus will cause the canal and otolith neurons to be activated at stimulus onset and show phase locking—firing one spike per cycle of the vibration stimulus [3]. A 100 Hz vibration stimulus causes an immediate large increase in primary neuronal firing rate which in TUVL patients would quickly drive the eyes away from the healthy side (slow phase) interspersed with QPs away from the affected ear. At stimulus offset there is a very abrupt return to resting activity [3] which causes an abrupt restoration of average VN neural activity and so an abrupt cessation of eye movements.

## 3. A Simplified Schematic Model of Nystagmus Generation

Based on physiological results, both neural and oculomotor, I propose the following simplified “imbalance principle” of nystagmus generation.

### Nystagmus Is Due to an Asymmetry of Average Neural Activity between the Two Vestibular Nuclei (VN)

Evidence for this “imbalance principle” has come from the results of recordings of guinea pig single neurons in the two vestibular nuclei (VN) after TUVL [14,15]. Acutely after TUVL there is a large imbalance in the average neural activity between the two VN, with the VN neurons on the lesioned side having a low or absent resting discharge, whereas neurons in the VN on the remaining healthy side having a normal or even elevated, average resting discharge. Thus, there is a major imbalance in average neural activity of the two VN. Corresponding to this neural imbalance, alert guinea pigs at this acute time after TUVL, show a strong nystagmus with quick phases beating away from the lesioned side [16]. The activity of the stronger VN drives the SPV in a direction opposite to the stronger VN and this SPV is interspersed with quick phases (QPs) directed away from the lesioned side. Over about 30 h after TUVL the asymmetry in average resting activity between the two VN progressively diminishes [14,15], and correspondingly, there is a progressive diminution of the nystagmus.

**Figure 3:**
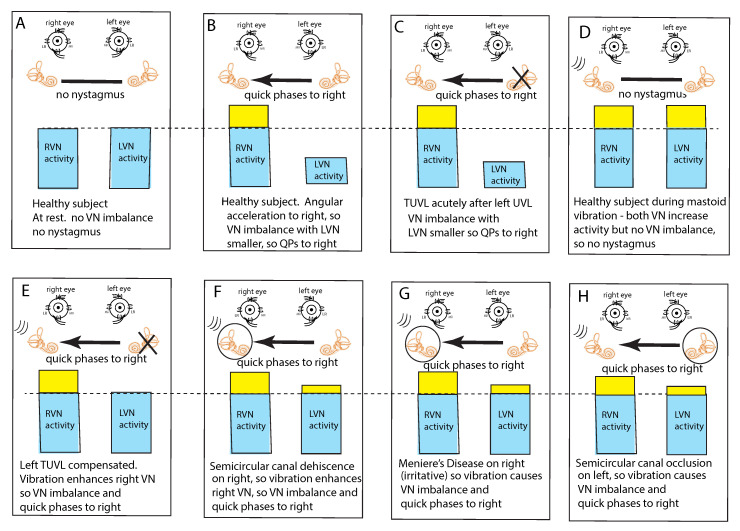
The “imbalance principle”—showing the relative average neural activity of the two vestibular nuclei (VN—histograms) in various stimulus conditions resulting in nystagmus with the direction shown by the black arrow. The yellow areas show the increased activity resulting from the procedure. These histograms are representations of the level of neural activity in the vestibular nuclei in eight separate conditions (**A**–**H**). The thin dashed line represents the average level of activation of a healthy subject at rest. The three lines (in **D**–**H**) indicate the vibration stimulus which, even though it is applied to only one labyrinth, is an effective stimulus to both labyrinths simultaneously because of the fast efficient conduction through the skull. In healthy people it is presumed that the average neural activity in the two nuclei is in balance (**A**) with no nystagmus. (**B**) A horizontal head angular acceleration to the right will activate the right VN and simultaneously decrease the activity of the left VN. This imbalance results in nystagmus with slow phase eye velocity away from the right side and quick phases towards the right side. An observer perceives the quick phases and so the nystagmus appears to beat towards the right side (and away from the left side). A patient acutely after left unilateral loss (**C**) has a large imbalance in neural activity so quick phases beat to the right. (**D**) If a healthy person is subject to vibration of either mastoid it will generate increased neural activity in both VN (**D**) because canal neurons in both labyrinths will be activated, but there will be no asymmetry in average VN neural activity and so no nystagmus. The circled labyrinths in (**F**–**H**) show the affected labyrinth.

In healthy subjects at rest, it is assumed that the average neural activity is about the same in both vestibular nuclei (VN) since there is no nystagmus (Figure 3A). If the average neural activity in one VN is greater than the other VN there will be a nystagmus with slow phases directed away from the VN with the larger average neural activity (the side of healthy ear) and QPs directed away from (opposite to) the side of the VN with the lower average neural activity (the affected ear). Such an asymmetry or imbalance or inequality in average neural activity can arise because of reduced input to the VN from the peripheral vestibular system of one ear or increased neural input to the other ear (Figure 3B,C). Conditions which can cause such an average imbalance are e.g., horizontal angular acceleration (Figure 3B) or unilateral vestibular loss (Figure 3C). Such an imbalance between the VN activity will result in nystagmus with quick phases beating away from the side with the lower average neural activity.

This VN imbalance principle also explains why mastoid vibration stimulation of either mastoid in healthy subjects does not cause nystagmus; mastoid vibration will activate canal receptors in both labyrinths simultaneously because of the very effective transmission of vibration across the skull [17], so both VN will have increased average neural activity but there will be no imbalance in average neural activity between the two VN and thus no nystagmus (Figure 3D). This simultaneous activation of both horizontal canals is a unique stimulus—it can never happen during natural head movements. Cohen et al. showed simultaneous bilateral electrical stimulation of both horizontal canal nerves in cats caused no horizontal eye movement [5] which they argued occurred because opposing eye muscles were simultaneously activated and so eye movement was cancelled.

This imbalance principle also explains why mastoid vibration causes SVIN after a patient with TUVL has compensated and so has minimal or absent spontaneous nystagmus (Figure 3E). That absence of nystagmus implies that the average neural activity in both VN is about equal. The 100 Hz unilateral mastoid vibration stimulus on the healthy side will activate semicircular canal receptors and irregular afferents projecting to and activating the VN on the healthy side. However, there is no afferent neural input from the opposite (lesioned) labyrinth, so there is an imbalance in average neural activity between the two VN resulting in nystagmus with QPs directed away from the lesioned side. Stimulation of the mastoid of the affected ear will cause the vibration stimulus (represented by three lines near right labyrinth in D–H) to be transmitted through the skull very effectively and so this contralateral vibration stimulation will activate the canal and otolith receptors on the remaining (healthy side) and so will also result in an average neural imbalance between the two VN and so SVIN in the same direction for stimulation of either mastoid.

Semicircular canal neurons show progressively poorer response to vibration as the vibration frequency is increased above 100 Hz (Figure 2D. However, a dehiscence of the semicircular canal (SCD) increases the fluid displacement caused by the stimulus [18]. so high vibration frequencies such as 500 Hz will generate fluid displacements large enough to deflect the cilia and so sufficient to activate previously unresponsive afferents at 500 Hz [3]. Semicircular canal neurons which were unresponsive to 500 Hz vibration with an intact bony labyrinth, respond vigorously to vibration at 500 Hz (and higher frequencies) after an experimental SCD [3,19]. Correspondingly human SCD patients show SVIN to frequencies higher than the 100 Hz cut off in TUVL patients with intact bony labyrinths [8].

This imbalance principle also explains why in some SCD patients (Figure 3F) and some patients with MD (Figure 3G), vibration causes a reverse SVIN with the quick phases of SVIN beating towards the affected ear (the ear with the SCD or MD—the labyrinth circled in Figure 3) instead of away from the affected ear as occurs after TUVL. As a result of the enhanced semicircular canal neural response to vibration after SCD, unilateral mastoid vibration of a patient with an SCD will generate an imbalance between the two VN but now the VN on the side of the ear with the SCD will have a higher average firing rate than VN on the opposite side so, according to the imbalance principle, the quick phases will be driven towards the ear with the SCD since the VN on that side has the higher average neural activity (Figure 3F).

A similar account probably applies in some cases of MD; in the paralytic phase, MD may reduce the response of afferent neurons in the affected ear (and so result in imbalance of VN activity and SVIN with QPs away from the affected ear), but in the irritative phase the disease may enhance the response of afferent neurons [20,21,22] and so result in nystagmus with QPs towards the affected ear. In both SCD and the irritative phase of MD, it is, on this account, an enhanced response which causes the imbalance in neural activity between the two VN and so drives QPs towards the affected ear (Figure 3G).

Physically blocking the membranous duct of a semicircular canal (called canal occlusion) reduces the capacity of the canal receptors and afferents to respond to angular acceleration stimulation and so the gain of the human vestibulo-ocular reflex is significantly attenuated [23,24,25]. It is highly likely that the occlusion of a fully encased semicircular canal also reduces the response to low frequency skull vibration (although definite evidence of the effect of canal occlusion on the response of primary canal afferents to 100 Hz vibration (with intact bony labyrinth) has not yet been reported). So, the mastoid vibration in patients with unilateral semicircular canal occlusion would be expected to be attenuated and so result in a neural imbalance at the level of the VN. which would lead to SVIN with QPs away from the side of the occluded canal as has been reported [6] (Figure 3H).

## 4. Clinical Value and Interpretation of SVIN

If a patient shows SVIN which is the same direction for 100 Hz vibration stimulation of both mastoids, it indicates that there is probably an asymmetry in semicircular canal function between the two labyrinths. The direction of the QPs indicates which labyrinth has the smaller response, since the nystagmus QPs beat away from the side with the smaller response. However, that nystagmus direction alone is ambiguous—the QPs beat away from the weaker side but that can happen because the VN activity of one side is reduced compared to the other or because the VN activity of one side is enhanced compared to the other. In both cases the nystagmus will beat away from the weaker side. Clinically these two conditions are diametrically opposed—in one case there is reduced function of one ear and in the other there is enhanced function of the opposite ear. This ambiguity is an especially pronounced problem in MD, where there can be either reduced or enhanced function even during a vertigo attack and the level of VN asymmetry probably varies during the vertigo attack [21,22] since the direction of the spontaneous nystagmus may reverse. Proper diagnostic interpretation of SVIN requires other clinical evidence in addition to the direction of QPs—e.g., evidence which independently indicates which is the affected side having lower function or enhanced function (e.g., spontaneous nystagmus, functional vestibular tests such as calorics or video head impulse testing, or low frequency hearing loss).

## 5. SVIN—The Slow Phase Eye Velocity

The Dumas group has made three-dimensional recordings of the eye movements induced by mastoid vibration, and many of their results can be explained by reference to the results from stimulation of isolated semicircular canals [5]. The recordings show that in TUVL patients SVIN typically has both horizontal and torsional components with the SPV of both components directed away from the healthy ear interspersed with QPs directed away from the affected ear. The neural evidence is that the mastoid vibration activates all semicircular canals (and otoliths) simultaneously [4], and combined with the evidence from Cohen and Suzuki’s canal stimulation, explains why there is horizontal, torsional, but not vertical, components in SVIN.

**Figure 4:**
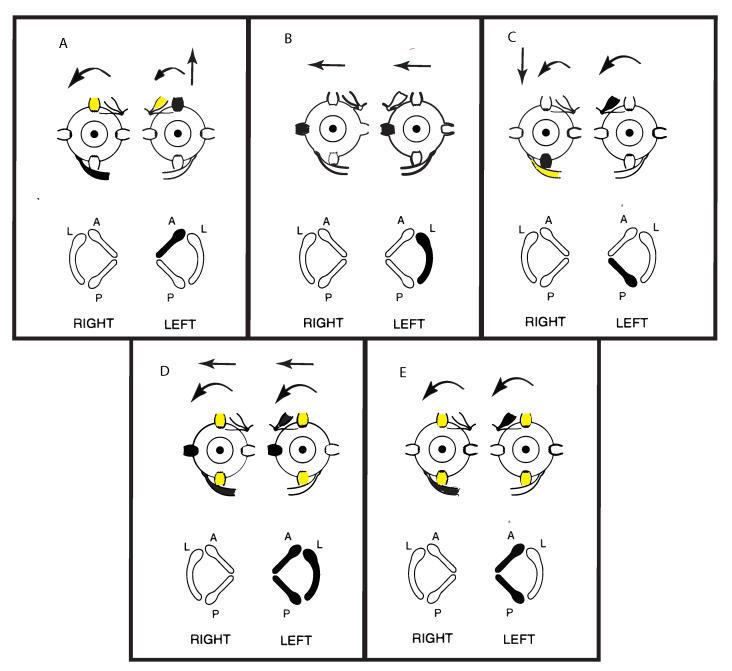
Representations of the direction of eye movements in response to selective electrical stimulation of individual canals (from Cohen, Suzuki, Bender 1964). The point of view of the semicircular canals in the schematic is straight down on the labyrinths. The arrows in these figures show the direction of the slow-phase eye velocity of the eye movement response to electrical stimulation of individual canals. The stimulated canal(s) are shown in black and the main eye muscles generating the eye movement (arrows) are shown as black. The muscles providing a smaller contribution are shown as yellow. (**A**–**C**) show the slow phase eye movements induced by stimulation of individual canals. Note that the movements in (**A**,**C**) are disconjugate, while those in (**B**) are conjugate horizontal eye movements away from the stimulated side. (**E**) shows the effect of simultaneous stimulation of both vertical canals in a labyrinth—generating torsion away from the stimulated ear, with minimal vertical or horizontal components. Simultaneous stimulation of all canals in a labyrinth (**D**) causes horizontal and torsional eye movements, with minimal vertical components. Reproduced from [5] with permission of Sage.

Cohen, Suzuki, and Bender conducted a pioneering series of experiments in cats where they used high frequency electrical stimulation by very fine bipolar stimulating electrodes to activate the nerves from each individual semicircular canal nerve selectively. They observed the directions of the SPV eye movement responses to such selective stimulation of individual canal nerves [5]. A schematic representation of some of their results is shown in Figure 4. They also simultaneously stimulated pairs of canal nerves and all three semicircular canals in one labyrinth and reported the eye movements.

Cohen et al. found that unilateral activation of neurons from the horizontal canal in one labyrinth (Figure 4B) caused horizontal eye movements with slow phases directed away from the stimulated side and QPs directed towards the stimulated side. These are the standard directions of the slow phase and QP directions of horizontal per-rotatory nystagmus. Stimulation of only the anterior canal or only the posterior canal both generated two components—a vertical as well as a torsional component. The vertical SPV component for the anterior canal was directed upward, and for the posterior canal the vertical SPV was directed downward, but the torsional SPV component was, in both cases, directed in a similar direction (away from the stimulated side) (Figure 4A,C). Simultaneous electrical stimulation of both anterior and posterior canal nerves within one labyrinth caused torsional eye movements directed away from the stimulated side, with minimal vertical components (Figure 4E). Cohen et al. reasoned that the simultaneous activation of the anterior and posterior canals in the one labyrinth results in the upward and downward vertical components cancelling (as shown by the simultaneous activation of opposing eye muscles). They had previously demonstrated that simultaneous stimulation of both horizontal canals results in such cancellation. However, with the vertical canals, each vertical canal generates a torsional component in the same direction (rolling away from the stimulated side) (Figure 4A,C). So, they argued that simultaneous stimulation of both vertical canals causes the opposite vertical components to cancel but the two torsional components to sum, because both torsional components are in the same direction (Figure 4E). If all three canals in a labyrinth were simultaneously stimulated (as the neural results indicates happens in SVIN) then there was a strong horizontal and torsional eye movement with minimal vertical component (Figure 4D). There is very close correspondence between these neural results and the eye movements of TUVL patients with SVIN, in particular the torsional as well as horizontal components.

## 6. Discussion

What is still unclear is the exact mechanism at the level of the receptor hair cell by which low frequency vibration deflects the stereocilia of the canal receptors sufficiently to activate them [26]. The vibration stimulus is a linear acceleration applied to the skull which may generate small fluid displacements which at frequencies of 100 Hz and below is sufficient to deflect the stereocilia of semicircular canal receptors. However, as the frequency is increased above 100 Hz the amplitude of the skull displacement decreases and so the fluid displacement will also decrease, and by 500 Hz the deflection of the stereocilia of the receptor hair cell is probably sub-threshold, at least in response to linear acceleration amplitudes which can be delivered with clinical safety to human subjects and patients. Canal occlusion probably also causes a similar reduction in fluid displacement.

In this paper I have focused on patients with known complete unilateral loss because it is in many ways the simplest case. Although, of course, many patients have partial loss of labyrinthine function or unknown problems and they can present with complex patterns of response to SVIN [1]. This paper shows how the responses of such patients with partial loss might be understood by considering the basic physiological evidence in relation to the “imbalance principle”.

The neural imbalance principle of nystagmus can also explain why vibration of neck muscles can induce nystagmus [27] since there are projections from neck muscle receptors to the vestibular nuclei [28,29,30] and so unilateral vibration of neck muscle can also produce an imbalance in average neural activity between VN in TUVL patients (and even healthy subjects), and so nystagmus [27].

Low frequency SVIN is a strong stimulus for irregular otolithic afferents [11] so it is possible (even likely) that there is otolithic contribution to the SVIN response. Otolith afferents converge on semicircular canal neurons in the vestibular nuclei [31,32] and otolithic stimulation of human observers can modulate the amplitude of the horizontal component of canal-induced nystagmus [33]. However there is a dissociation between clinical otolithic tests (such as VEMPs) and SVIN [34] indicating that while otolithic stimulation may be able to modulate SVIN, it is not (usually) the main cause of the SVIN.

## Figures and Tables

**Figure 1:**
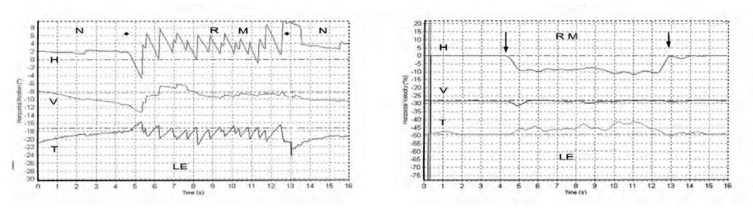
Left panel. Horizontal, vertical, and torsional eye position of a TUVL patient during skull vibration. The onset and offset of the skull vibration are shown by asterisks. The horizontal and torsional components start at stimulus onset and cease at offset. The records show the horizontal, vertical and torsional eye movement recordings (Synapsys) of a TUVL patient with a total left-sided vestibular loss in response to stimulation of the right mastoid (RM). The vibration stimulus causes horizontal and torsional quick phases to the right, away from the affected left ear. In the Synapsys recording conventions, a horizontal quick phase to the right is shown by an upwards deflection of the trace and a torsional quick phase to the right (with the upper pole of the eye rolling to the patient’s right side) is shown by a downward deflection of the trace. The horizontal (and torsional) quick phases are directed away from the lesioned ear. There is no consistent vertical component. Right panel. The slow phase eye velocities corresponding to the eye position records. From [1] with permission.

**Table 1:** A summary of the main characteristics of the SVIN response of patients with total unilateral vestibular loss (TUVL) summarized from [1] and related papers by the Dumas group.

Main Clinical Results from SVIN Studies of the Response of Patients with Total Unilateral Vestibular Loss (TUVL)
In response to 100 Hz mastoid vibration there is a stimulus-locked nystagmus with quick phases beating away from the lesioned ear
2. The direction of the nystagmus is predominantly horizontal together with a torsional component, but rarely a vertical component
3. The direction of the SVIN is the same for vibration of either mastoid
4. The SVIN is time-locked to the stimulus with an abrupt onset and an abrupt offset locked to the onset and offset of the vibration stimulus (with no afternystagmus)
5. The slow phase velocity of the nystagmus increases as vibrator stimulus frequency is increased from 30 Hz up to 100 Hz, however at frequencies above 100 Hz the SPV declines so that in TUVL patients a stimulation frequency of 500 Hz at clinically acceptable and safe stimulus levels is ineffective in generation of SVIN
6. Comparable mastoid stimulation procedures in healthy subjects fail to produce reliable SVIN with slow phase velocities greater than about 2.5 deg/s
**Other major results**
7. In patients with one horizontal semicircular canal occluded, the QPs of the SVIN typically beat away from the side of the occluded canal [6], just as for TUVL patients 8. In patients with a semicircular canal dehiscence (SCD), mastoid vibration can induce a reverse SVIN: with quick phases directed toward the ear with the SCD [7,8].
9. In some patients with Menière’s Disease (MD) there can be similar reverse nystagmus with quick phases directed toward the affected ear [1].

## Data Availability

Not applicable.

## Abbreviations

MD	Menière’s Disease
QP	quick phases
SCD	semicircular canal dehiscence
SPV	slow phase eye velocity
SVIN	skull vibration induced nystagmus
TUVL	total unilateral vestibular loss
VN	vestibular nuclei
